# SPIEDw: a searchable platform-independent expression database web tool

**DOI:** 10.1186/1471-2164-14-765

**Published:** 2013-11-07

**Authors:** Gareth Williams

**Affiliations:** Wolfson Centre for Age-Related Diseases, King’s College London, London Bridge, London, SE1 1UL UK

**Keywords:** Global gene expression, Connectivity map, Microarray, EGF receptor, Parkinson's disease

## Abstract

**Background:**

SPIEDw is a web tool designed to facilitate fast and simple quantitative querying of publically available gene expression data. The resource is motivated by the observation that transcriptional profiles can serve as effective means of comparing biological states across a wide set of experiments.

**Results:**

Gene expression data for over 200,000 experiments across multiple species and platforms have been collected into a searchable database. The new resource is a development of the previously published SPIED, which was designed to be downloaded and queried locally with SPIED software. SPIEDw features three significant improvements over the original version. Firstly, the number of experiments covered has been doubled and now includes Agilent and Illumina technologies. Secondly, SPIEDw has an enhanced search algorithm for speedy web-based querying and lastly an abridged dataset comprising the most regulated genes has been included for a speedier search and searching for enrichment of gene sets.

**Conclusions:**

SPIEDw is simple to use, not requiring any expertise in microarray analysis, and the output straightforward to interpret. It is hoped that this will open up gene expression data mining to the wider research community.

**Electronic supplementary material:**

The online version of this article (doi:10.1186/1471-2164-14-765) contains supplementary material, which is available to authorized users.

## Background

Gene expression data provides the most extensive quantitative insight into biological processes with close to a million separate data sets available [1, 2]. The data is usually analysed from a gene centric point of view, with the objective of pinpointing a few perturbed genes that are subsequently independently validated. Alternatively, expression profiles can be viewed as the regulation of predefined gene sets representing canonical pathways [3] and ontology classes [4, 5]. Various web tools allow for the navigation of deposited expression data. BioGPS [6] and SOURCE [7] are tools for gene comparison based on correlating expression profiles over large datasets. ArrayExpress Gene Expression Atlas [8] has compiled a database of significantly changing genes across nearly 100,000 expression samples and the user can search for expression series where a query gene or gene set are regulated. However, the observation that similar biological processes often exhibit correlated gene expression changes has opened up the possibility of using expression databases to compare and contrast these processes [9, 10]. In particular, the connectivity map (CMAP) has made available expression data for over a thousand drug treatments of human cell lines with the objective of mapping drug to disease through direct expression profile correlation [11]. This methodology has been extended to collections of published expression studies in GEM-TREND [12], ProfileChaser [13], Gene Expression Atlas [8] and SPIED [14]. The motivation behind SPIED was in the first place to define expression changes as relative to the average across the experimental series and thereby facilitate automatic curation and secondly to map expression changes to a uniquely defined set of genes thus allowing for the database to combine data from multiple platforms. The original version of SPIED has to be downloaded and searches performed locally with SPIED software. Here we present a web tool version of SPIED (SPIEDw). SPIEDw features a speedier search algorithm enabling 'real-time’ web-based searching and consists of a greatly extended set of samples covering human, mouse and rat species from Illumina, Agilent and Affymetrix platform technologies, comprising half the available data for these species. In addition to exhaustive querying of global gene expression changes, a speedier search can be made of an abridged database comprising the most regulated genes. This latter functionality can also be deployed for gene set enrichment analysis.

## Results

**Figure 1:**
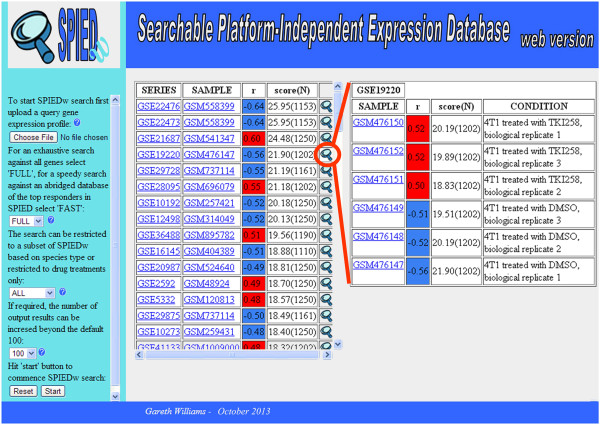
**A SPIEDw worked example.** The SPIEDw web page screen shot showing the various controls in the left column. A gene expression profile is uploaded, the user chooses whether to perform an exhaustive search or search only an abridged dataset comprising the most regulated genes. The latter is the appropriate option for performing gene list enrichment queries. Searches can be restricted to specific species and the number of top scoring samples returned can be increased beyond the default 100. We have performed a full search of SPIEDw with a profile corresponding to EGFR blockage in mouse neural stem cells [15]. The results column at the left lists the series id and sample id together with the correlation score and significance. The 'magnifying glass’ button enables the user to see how the query scores against all the samples in the given series, displayed in the results column on the right. For example, the EGFR antagonism profile scores highly against a study of PI3K/AKT inhibition in mouse mammary carcinoma cells [16], and as expected, this correlation is positive with the inhibitor treated samples and negative with the control samples.

SPIEDw is hosted at http://www.spied.org.uk. Query expression profiles consist of text files with the first two columns corresponding to genes and their respective expression values. Gene names are according to the HGNC (HUGO Gene Nomenclature Committee) human gene nomenclature, see http://www.genenames.org. Given that the overwhelming majority of gene names are identical across human, mouse and rat queries can be constructed directly from rodent array data files. Queries should not contain multiple instances of genes. When a query is input containing multiple instances of a given gene only the first instance is retained in the query. Therefore, if the query is ranked according to significance only the most significant instances will form the query. Expression values can be defined in a number of ways, but treat up and down regulation symmetrically. Possible definitions are: , , , where *t* is the treatment and *c* the control values. The database can either be queried exhaustively against the global gene changes or against subsets of top up and down regulated genes. Querying the latter database has the advantage of being much faster and unlike the global gene expression database it can be meaningfully queried with gene lists alone where it is of interest to look for enrichment for up or down regulation across experiments in SPIEDw. Searches can be restricted to a particular species (Human, Rodent, Mouse, Rat) or performed against the CMAP drug dataset. The output is in the form of a list of series names, sample names and corresponding scores. The output lists the top scoring samples and only one sample per series. The series and sample entries link to the relevant NCBI GEO (http://www.ncbi.nlm.nih.gov/geo/) pages giving full descriptions of the experimental set up. To see how the query scores against the whole series, from which a high scoring sample originates, there is a 'magnifying glass’ button link against each output sample. The query scores against each sample in the series are ordered according to correlation score and the associated descriptors are shown. This is a simple way to see a possible correspondence between the biology underlying the series and that of the query. For clarity, the SPIEDw web interface is shown in Figure 1 together with a worked example. With the current server configuration full searches take just over four minutes.

**Figure 2:**
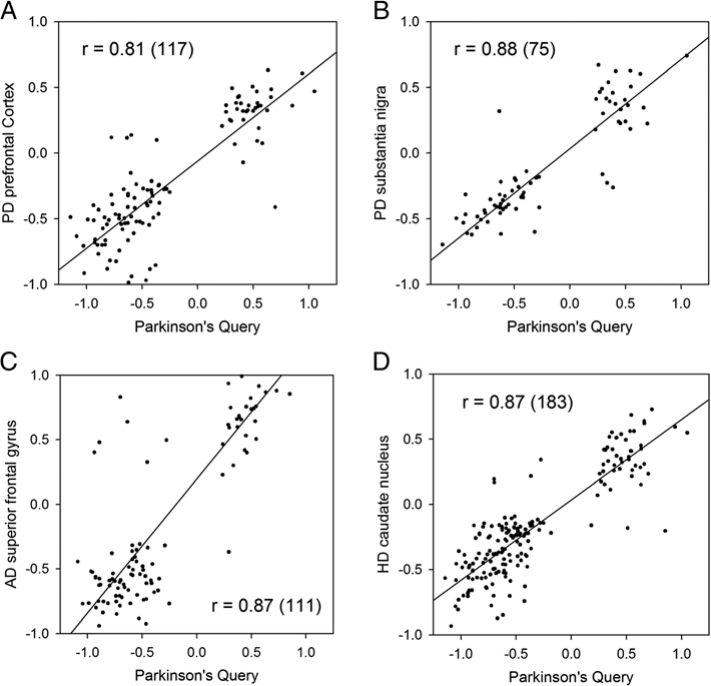
**The regression plots for the PD query profile against high scoring PD, AD and HD expression studies in SPIEDw.** The regression plots are shown for high scoring PD expression profiles derived from post-mortem brain samples from two independent publications. In **A** the PD query is plotted against a PD profile from the prefrontal cortex [20] and in **B** against a PD profile from the substantia nigra [21]. The PD query also scores highly against other neurodegenaration disease profiles and two example regression plots are shown in **C**, for an AD profile from the superior frontal gyrus [22], and **D**, a HD profile from the caudate nucleus [23].

Examples of the use of SPIED in diverse disease contexts were presented in some detail in the original publication [14]. However, for completeness we present an example where SPIEDw can be used to validate transcriptional signatures from Parkinson’s (PD) disease and shed light on animal models of PD. This complements the analysis of Alzheimer’s disease (AD) presented in [14]. The notorious difficulty of obtaining good quality RNA from post-mortem brain samples [17, 18] raises legitimate concerns over the robustness of neurodegenerative disease associated transcription signatures. However, when there is a significant correlation with independently derived disease profiles in the public domain we can be more confident that these profiles describe the disease. SPIEDw offers a straightforward application here. To this end we took a PD signature form a multi-regional brain expression study [19]. By combining the gene change profiles form the three regions we defined a profile of 235 genes (67 up and 168 down, see Additional file 1 for details). When this profile is queried against the human subset of SPIEDw we find nine independent PD profiles in the top 100 experimental series, see Table 1. Thus these results go some way towards validating the profile. Profiles from other neurodegenerative conditions also score highly, see Table 2. Because the given expression series consists of samples from multiple brain areas with corresponding controls, a better idea of the disease correlation can be seen between statistically filtered expression profiles based on grouping control and disease sets. Example regression plots are shown in Figure 2A and B and it is clear that there is a substantial overlap between the PD query and two independent PD profiles. This correlation is also seen with profiles corresponding to AD and Huntington’s diseases (HD), see Table 3 and the two regression plots in Figure 2C and D. When the PD profile is queried against the rodent subset of SPIEDw we find that there is a significant correlation with rodent models of neurodegeneration, see Table 4, and in particular a mouse model of PD based on the administration of MPTP (GEO accession GSE7707). This is in contrast to the AD situation, where there doesn’t appear to be any significant correlation with animal models of the disease (GW unpublished observation). Again to get a better idea of the overlap of the query profile with the rodent model profiles we generated statistically filtered profiles for the various conditions and generated regression scores. These are shown in Table 4. Interestingly, the PD correlation with the MPTP model only holds up in the striatum as opposed to the midbrain or frontal cortex. Also, a significant correlation emerges with late stage profiles of the spinal chord injury model and the SOD1(G93A) model of Amyotropic lateral sclerosis (ALS) (GEO accession GSE18597), a similar result to previously published AD query results [14]. The expression profiles used in the regression analyses together with the PD query profile are given in the Additional file 1.

**Table 1:** **The PD query profile scores highly against nine other PD expression studies, thus validating the query as a disease signature**

Parkinson’s disease			
Rank	Series	r	Description
1	GSE8397	-0.92	Parkinsonian Brain
3	GSE20168	-0.88	Prefrontal area 9 in Parkinson’s disease
13	GSE7621	-0.82	Substantia nigra from postmortem human brain of Parkinson’s disease
21	GSE28894	-0.78	Multiple regions of the Parkinson’s disease brain
58	GSE20141	-0.62	Laser-dissected SNpc neurons in Parkinson’s disease
71	GSE4550	0.6	MPTP-treated macaques
89	GSE20292	-0.55	Substantia nigra in Parkinson’s disease
97	GSE19587	-0.53	Parkinson’s disease
99	GSE20291	-0.51	Putamen in Parkinson’s disease

**Table 2:** **Parkinson’s disease query correlates with the expression profiles of multiple neurodegenerative expression**

Frontal temporal lobe dementia			
Rank	Series	r	Description
2	GSE13162	0.92	Samples with and without FTLD-U
**Huntington’s disease**			
**Rank**	**Series**	**r**	**Description**
5	GSE3790	0.84	Cerebellum, frontal cortex and caudate nucleus HD tissue
**Down’s syndrome**			
**Rank**	**Series**	**r**	**Description**
7	GSE5390	0.84	Down syndrome and healthy control subjects
86	GSE1397	0.55	Trisomy 21 and TS13
**Bipolar disorder**			
**Rank**	**Series**	**r**	**Description**
10	GSE5388	0.83	Adult dorsolateral prefrontal cortex: bipolar disorder and healthy controls
11	GSE5389	0.83	Adult orbitofrontal cortex: bipolar disorder and healthy controls
15	GSE5392	0.82	Adult brain tissue from subjects with bipolar disorder and healthy controls
**Alzheimer’s disease**			
**Rank**	**Series**	**r**	**Description**
14	GSE16759	-0.82	Parietal lobe cortex in Alzheimer’s disease
26	GSE1297	-0.75	Incipient Alzheimer’s Disease
38	GSE5281	0.69	Alzheimer’s disease and the normal aged brain
56	GSE12685	-0.63	Incipient AD
68	GSE4757	0.61	Alzheimer’s disease neurofibrillary tangles
**Nasu-Hakola disease**			
**Rank**	**Series**	**r**	**Description**
33	GSE25496	0.73	Nasu-Hakola disease brain
**Multiple sclerosis**			
**Rank**	**Series**	**r**	**Description**
40	GSE5839	0.69	Multiple Sclerosis Brain tissue in comparison to control brain tissue
**Amyotropic lateral sclerosis**			
**Rank**	**Series**	**r**	**Description**
54	GSE4595	-0.66	Motor cortex in sporadic amyotrophic lateral sclerosis

**Table 3:** **The PD query correlates with the expression profiles of rodent models of neurodegeneration**

Amyotrophic lateral sclerosis (ALS) model			
Rank	Series	r	Description
12	GSE18597	0.6	Mutant SOD1(G93A) transgenic mice
**Ischemia**			
**Rank**	**Series**	**r**	**Description**
44	GSE23163	0.56	Ischemic/reperfusion injury in an *in vivo* mouse model
49	GSE23162	0.55	Ischemic/reperfusion injury in an *in vivo* Gpx1 -/- transgenic mouse model
92	GSE28201	0.44	Ischemic Mouse Brain Transcriptome
93	GSE33725	0.44	Effect of erythropoietin on cerebral ischemia in rat
**Parkinson’s model**			
**Rank**	**Series**	**r**	**Description**
55	GSE7707	-0.49	Mouse MPTP model of Parkinson’s disease
**Spinal chord injury**			
**Rank**	**Series**	**r**	**Description**
98	GSE5296	0.42	Spinal Cord Injury Murine Model
**Other**			
**Rank**	**Series**	**r**	**Description**
13	GSE18356	-0.61	Rat model of neonatal HI encephalopathy +/- antioxidant
19	GSE24131	0.58	Hypoxia effect on mouse neural stem cells
41	GSE14802	0.52	Smith-Magenis and Potocki-Lupski syndrome mouse model
54	GSE30577	-0.49	Brain and Spinal cord following Lethal CVS-11 Infection in Mice
87	GSE25250	-0.44	Cerebellum from mice exposed to chronic low-level chlorpyrifos oxon
88	GSE12196	0.53	Rat exposure to RDX

**Table 4:** **The regression scores for the PD query profile against mouse models of neurodegeneration**

MPTP mouse PD model		
Brain region	r	N
Striatum	0.39	56
Midbrain	0.08	32
Frontal cortex	-0.19	31
**SOD1 (G93A) mouse ALS model**		
**stage (days)**	**r**	**N**
28-40	0.21	63
112-126	0.48	104
**Mouse spinal chord injury**		
**stage**	**r**	**N**
24 h	0.33	57
28 days	0.57	65

## Discussion and conclusions

The motivation behind SPIEDw is to facilitate the simple quantitative comparison of biological states through their underlying gene transcription profiles. In comparison to the previously published SPIED, the new database has three notable improvements. Firstly, an enhanced search algorithm has enabled queries to be performed in 'real time’ on the web. Secondly, the number of expression samples comprising the database has been doubled, now covering Illumina and Agilent as well as Affymetrix platform technologies. Lastly, an abridged dataset comprising the most regulated genes has been included for a speedier search and for gene set enrichment analysis. The tool is simple to use, with queries consisting only of gene names and expression changes. The output is straightforward to interpret, giving direct links to the deposited data for the high scoring SPIEDw entries. It is hoped that this will open up gene expression data mining to the wider research community. At present SPIEDw consists of human and rodent arrays, where there is a great deal of homology and mostly unambiguous gene assignment. However, there have been extensive gene expression studies in more distant species, such as drosophila. It is hoped to extend SPIEDw in this direction with appropriate gene lists.

## Methods

Gene expression sample files were downloaded from NCBI GEO (http://www.ncbi.nlm.nih.gov/geo/) covering human, mouse and rat species on multiple platforms based on Affymetrix, Illumina and Agilent technologies. In particular the platforms are: Affymetrix {GPL570, GPL96, GPL6244, GPL571, GPL1261, GPL81, GPL6246, GPL85, GPL1355}; Illumina {GPL6947, GPL6104, GPL6883, GPL6102, GPL10558, GPL6884, GPL6887, GPL6885, GPL6105}; Agilent {GPL4133, GPL6480, GPL1708, GPL4134, GPL7202, GPL4135, GPL7294, GPL2877, GPL890}. As in the original version of SPIED, the array probe sets are mapped onto HGNC human genes. Each sample entry of SPIEDw consists of a list of genes ranked according to relative expression level in the given series to which the sample belongs. In addition an abridged database with individual sample entries corresponding to the top 500 most up/down regulated genes has been generated for fast querying. Here the top 500 up/down regulated genes are assigned only up/down status and query correlations are based on a Fisher exact test, see below. This database can also be queried for gene set enrichment analysis. Drug treatment data was downloaded from the Broad connectivity map site and here multiple drug instances were combined to obtain average response profiles and the SPIEDw entries for these consist of the ranked list of significantly affected genes (*p* < 0.05 by Student’s *t*-test) according to fold magnitude.

For full and drugs set searches query profiles are scored according to the significance of the Pearson’s correlation. In particular the ranking score is , where *r* is the Pearson’s correlation coefficient and *N* is the number of genes in the given correlation. To enable the user to make an assessment of the significance of the output, *r*, *s* and *N* are provided for each sample. The abridged database search is based on a Fisher exact test, where the given distribution of query genes in each database entry is compared to a random distribution and scored accordingly. Here, the separate enrichment scores for the up and down regulated genes are combined for the ranking score. Therefore a meaningful significance score will result from queries without fold change information. That is, the abridged database search can be used to look for enrichment of gene sets. In this case the query consists of a gene list with folds set to unity, see Additional file 2 for details.

To speed up database querying the data has been compressed into a base 150 format. Here the overwhelming majority of gene numbers are represented by no more than two indices, corresponding to ASCII characters. This results in a compression ratio of 59%. Data is read in blocks of 1000 arrays and processed. Note that converting the read buffers to integers is as fast for the base 150 data as for the uncompressed data.

### Availability of SPIED

SPIEDw is available at http://www.spied.org.uk. Current version is 2.0 and new builds will become available in due course, incorporating more recent expression data.

## Electronic supplementary material

Additional file 1: **Expression profile details for queries used in Results analysis together with corresponding correlating profiles from SPIEDw.** Excel spread sheet. with gene names, fold changes and p-values for transcripts with significantly altered expression levels for the example PD query and correlating profiles from PD, AD, HD human samples. Correlating profiles form a mouse MPTP model of PD together with a mouse SOD1(G93A) model of ALS and a mouse model of spinal chord injury are also given. (XLS 2 MB)

Additional file 2: **Example of SPIEDw with gene set query.** A worked example with a query consisting of a gene set. Querying the abridged database ('FAST’ mode query) enables the user to discover SPIEDw response profiles with significantly enriched query gene sets. (DOC 98 KB)

## References

[1] Barrett T, Troup DB, Wilhite SE, Ledoux P, Rudnev D, Evangelista C, Kim IF, Soboleva A, Tomashevsky M, Edgar R (2007). NCBI GEO: mining tens of millions of expression profiles--database and tools update. Nucleic Acids Res.

[2] Parkinson H, Sarkans U, Kolesnikov N, Abeygunawardena N, Burdett T, Dylag M, Emam I, Farne A, Hastings E, Holloway E (2011). ArrayExpress update--an archive of microarray and high-throughput sequencing-based functional genomics experiments. Nucleic Acids Res.

[3] Subramanian A, Tamayo P, Mootha VK, Mukherjee S, Ebert BL, Gillette MA, Paulovich A, Pomeroy SL, Golub TR, Lander ES (2005). Gene set enrichment analysis: a knowledge-based approach for interpreting genome-wide expression profiles. Proc Natl Acad Sci USA.

[4] Zeeberg BR, Feng W, Wang G, Wang MD, Fojo AT, Sunshine M, Narasimhan S, Kane DW, Reinhold WC, Lababidi S (2003). GoMiner: a resource for biological interpretation of genomic and proteomic data. Genome Biol.

[5] Ashburner M, Ball CA, Blake JA, Botstein D, Butler H, Cherry JM, Davis AP, Dolinski K, Dwight SS, Eppig JT (2000). Gene ontology: tool for the unification of biology. The gene ontology consortium. Nat Genet.

[6] Wu C, Orozco C, Boyer J, Leglise M, Goodale J, Batalov S, Hodge CL, Haase J, Janes J, Huss JW (2009). BioGPS: an extensible and customizable portal for querying and organizing gene annotation resources. Genome Biol.

[7] Diehn M, Sherlock G, Binkley G, Jin H, Matese JC, Hernandez-Boussard T, Rees CA, Cherry JM, Botstein D, Brown PO (2003). SOURCE: a unified genomic resource of functional annotations, ontologies, and gene expression data. Nucleic Acids Res.

[8] Kapushesky M, Adamusiak T, Burdett T, Culhane A, Farne A, Filippov A, Holloway E, Klebanov A, Kryvych N, Kurbatova N (2012). Gene expression atlas update--a value-added database of microarray and sequencing-based functional genomics experiments. Nucleic Acids Res.

[9] Marton MJ, DeRisi JL, Bennett HA, Iyer VR, Meyer MR, Roberts CJ, Stoughton R, Burchard J, Slade D, Dai H (1998). Drug target validation and identification of secondary drug target effects using DNA microarrays. Nat Med.

[10] Hughes TR, Marton MJ, Jones AR, Roberts CJ, Stoughton R, Armour CD, Bennett HA, Coffey E, Dai H, He YD (2000). Functional discovery via a compendium of expression profiles. Cell.

[11] Lamb J, Crawford ED, Peck D, Modell JW, Blat IC, Wrobel MJ, Lerner J, Brunet JP, Subramanian A, Ross KN (2006). The connectivity Map: using gene-expression signatures to connect small molecules, genes, and disease. Science.

[12] Feng C, Araki M, Kunimoto R, Tamon A, Makiguchi H, Niijima S, Tsujimoto G, Okuno Y (2009). GEM-TREND: a web tool for gene expression data mining toward relevant network discovery. BMC Genomics.

[13] Engreitz JM, Chen R, Morgan AA, Dudley JT, Mallelwar R, Butte AJ (2011). ProfileChaser: searching microarray repositories based on genome-wide patterns of differential expression. Bioinformatics.

[14] Williams G (2012). A searchable cross-platform gene expression database reveals connections between drug treatments and disease. BMC Genomics.

[15] Sutterlin P, Williams EJ, Chambers D, Saraf K, von Schack D, Reisenberg M, Doherty P, Williams G (2013). The molecular basis of the cooperation between EGF, FGF and eCB receptors in the regulation of neural stem cell function. Mol Cell Neurosci.

[16] Dey JH, Bianchi F, Voshol J, Bonenfant D, Oakeley EJ, Hynes NE (2010). Targeting fibroblast growth factor receptors blocks PI3K/AKT signaling, induces apoptosis, and impairs mammary tumor outgrowth and metastasis. Cancer Res.

[17] Barton AJ, Pearson RC, Najlerahim A, Harrison PJ (1993). Pre- and postmortem influences on brain RNA. J Neurochem.

[18] Webster MJ (2006). Tissue preparation and banking. Prog Brain Res.

[19] Moran LB, Duke DC, Deprez M, Dexter DT, Pearce RK, Graeber MB (2006). Whole genome expression profiling of the medial and lateral substantia nigra in Parkinson’s disease. Neurogenetics.

[20] Zhang Y, James M, Middleton FA, Davis RL (2005). Transcriptional analysis of multiple brain regions in Parkinson’s disease supports the involvement of specific protein processing, energy metabolism, and signaling pathways, and suggests novel disease mechanisms. Am J Med Genet B Neuropsychiatr Genet.

[21] Lesnick TG, Papapetropoulos S, Mash DC, Ffrench-Mullen J, Shehadeh L, de Andrade M, Henley JR, Rocca WA, Ahlskog JE, Maraganore DM (2007). A genomic pathway approach to a complex disease: axon guidance and Parkinson disease. PLoS Genet.

[22] Liang WS, Dunckley T, Beach TG, Grover A, Mastroeni D, Walker DG, Caselli RJ, Kukull WA, McKeel D, Morris JC (2007). Gene expression profiles in anatomically and functionally distinct regions of the normal aged human brain. Physiol Genomics.

[23] Hodges A, Strand AD, Aragaki AK, Kuhn A, Sengstag T, Hughes G, Elliston LA, Hartog C, Goldstein DR, Thu D (2006). Regional and cellular gene expression changes in human Huntington’s disease brain. Hum Mol Genet.

