# Supplementary material for: SPIEDw: a searchable platform-independent expression database web tool
Source: BMC Genomics. 2013 Nov 7;14(1):765. doi: 10.1186/1471-2164-14-765 (PMC4046673; doi:10.1186/1471-2164-14-765)
Supplement: Supplementary file 2 — Additional file 2: Example of SPIEDw with gene set query. A worked example with a query consisting of a gene set. Querying the abridged database ('FAST’ mode query) enables the user to discover SPIEDw response profiles with significantly enriched query gene sets. (DOC 98 KB) [file 12864_2013_5470_MOESM2_ESM.doc]

**An example of the use of SPIEDw for a gene set enrichment query**

In addition to querying with gene expression data, SPIEDw allows for queries based on gene sets alone. This is provided for by the abridged version of SPIEDw, consisting of the top sliced up and down regulated genes. As the scoring in this case is based on the separate enrichment of the up and down regulated query genes, this database can effectively be queried with a profile consisting of only up regulated genes i.e. a gene set. In practice, a gene set is made into a SPIEDw query by assigning a fold of unity to each gene in the list. As an example we present the results based on a pathway gene set from the GSEA dataset. In particular, we took the G1_TO_S_CELL_CYCLE pathway consisting of 68 genes, the list is shown in additional file 1. Selecting the SPIEDw ‘FAST’ search option we get the following output:

| **SERIES** | **SAMPLE** | **correl** | **ln(prob)(N)** |
| --- | --- | --- | --- |
| GSE22886 | GSM565277 | 1.00 | -21.95(27) |
| GSE13710 | GSM344715 | -0.93 | -20.03(28) |
| GSE30597 | GSM758989 | -1.00 | -17.28(22) |
| GSE19274 | GSM478220 | -1.00 | -17.28(22) |
| GSE30392 | GSM754239 | -0.92 | -17.27(25) |
| GSE15208 | GSM379767 | 0.86 | -16.90(27) |
| GSE13041 | GSM326909 | 0.92 | -16.39(24) |
| GSE23038 | GSM568609 | 0.92 | -16.39(24) |
| GSE27260 | GSM673850 | 0.92 | -16.39(24) |
| GSE19123 | GSM474317 | 1.00 | -16.38(21) |
| GSE26549 | GSM652772 | 1.00 | -16.38(21) |
| GSE14034 | GSM352358 | 1.00 | -16.38(21) |

The top scoring sample is from a series of arrays of resting and activated human immune cells. The query gene set can be scored against all the samples in the series and this is achieved by clicking on the ‘magnifying glass’ link. As can be seen below there is a clear positive enrichment of the cell cycle gene set for the samples corresponding to activated cells and vice versa for the resting cells.

| **GSE22886** |  |  |  |  |  |  |
| --- | --- | --- | --- | --- | --- | --- |
| **SAMPLE** | **correl** | **ln(prob)(N)** | **CONDITION** | |  |  |
| GSM565277 | 1.00 | -21.95(27) | CD4Tcell-Th2-restimulated12hour-1 [HG-U133B] | | | |
| GSM565279 | 1.00 | -20.99(26) | CD4Tcell-Th2-restimulated12hour-1 [HG-U133A] | | | |
| GSM565285 | 1.00 | -19.11(24) | MemoryTcell-RO-activated-2 [HG-U133A] | | | |
| GSM565286 | 1.00 | -18.19(23) | CD4Tcell-Th2-restimulated48hour-3 [HG-U133A] | | | |
| GSM565290 | 1.00 | -17.28(22) | MemoryTcell-RO-activated-1 [HG-U133B] | | | |
| GSM565283 | 1.00 | -16.38(21) | CD4Tcell-Th2-restimulated12hour-3 [HG-U133A] | | | |
| GSM565292 | 1.00 | -16.38(21) | NKcell-control-2 [HG-U133A] | | |  |
| GSM565280 | 1.00 | -15.50(20) | CD4Tcell-Th1-restimulated48hour-3 [HG-U133A] | | | |
| GSM565302 | 1.00 | -15.50(20) | NKcell-IL15stimulated-1 [HG-U133A] | | | |
| GSM565297 | 1.00 | -15.50(20) | NKcell-control-3 [HG-U133B] | | |  |
| GSM565291 | 1.00 | -14.63(19) | MemoryTcell-RO-unactivated-3 [HG-U133B] | | | |
| GSM565282 | 1.00 | -14.63(19) | MemoryTcell-RO-unactivated-2 [HG-U133A] | | | |
| GSM565301 | 1.00 | -12.93(17) | NKcell-IL15stimulated-6 [HG-U133A] | | | |
| GSM565276 | 1.00 | -11.26(15) | CD4Tcell-Th1-restimulated12hour-1 [HG-U133A] | | | |
| GSM565303 | 1.00 | -10.45(14) | NKcell-IL2stimulated-4 [HG-U133B] | | | |
| GSM565281 | 1.00 | -9.64(13) | CD4Tcell-Th1-restimulated48hour-2 [HG-U133B] | | | |
| GSM565307 | 1.00 | -9.64(13) | NKcell-IL15stimulated-4 [HG-U133B] | | | |
| GSM565298 | 1.00 | -8.85(12) | NKcell-IL15stimulated-3 [HG-U133A] | | | |
| GSM565284 | 1.00 | -5.76(8) | CD4Tcell-Th2-restimulated48hour-1 [HG-U133B] | | | |
| GSM565304 | 0.91 | -13.82(21) | NKcell-IL15stimulated-1 [HG-U133B] | | | |
| GSM565306 | 0.90 | -12.18(19) | NKcell-IL15stimulated-2 [HG-U133B] | | | |
| GSM565278 | 0.82 | -11.13(20) | CD4Tcell-Th1-restimulated12hour-1 [HG-U133B] | | | |
| GSM565299 | 0.76 | -7.51(15) | NKcell-IL2stimulated-3 [HG-U133A] | | | |
| GSM565379 | -0.50 | -3.02(9) | Neutrophil-Resting-2 [HG-U133A] | | | |
| GSM565346 | -0.54 | -3.50(10) | Monocyte-Day1-11 [HG-U133A] | | |  |
| GSM565347 | -0.57 | -4.01(11) | Monocyte-Day1-6 [HG-U133A] | | |  |
| GSM565350 | -0.62 | -5.11(13) | Monocyte-Day1-9 [HG-U133A] | | |  |
| GSM565344 | -0.64 | -3.77(9) | Monocyte-Day1-3 [HG-U133A] | | |  |
| GSM565342 | -0.64 | -3.77(9) | Monocyte-Day0-11 [HG-U133B] | | |  |
| GSM565375 | -0.64 | -3.77(9) | Neutrophil-Resting-1 [HG-U133A] | | | |
| GSM565345 | -0.64 | -3.77(9) | Monocyte-Day1-4 [HG-U133B] | | |  |
| GSM565357 | -0.75 | -3.58(7) | Monocyte-Day7-4 [HG-U133A] | | |  |
| GSM565358 | -0.75 | -3.58(7) | Monocyte-Day7-3 [HG-U133B] | | |  |
| GSM565309 | -0.75 | -3.58(7) | Bcell-naÃ¯ve-3 [HG-U133B] | | |  |
| GSM565332 | -0.75 | -3.58(7) | Monocyte-Day0-3 [HG-U133B] | | |  |
| GSM565361 | -0.75 | -3.58(7) | Monocyte-Day7-8 [HG-U133B] | | |  |
| GSM565364 | -0.75 | -3.58(7) | Monocyte-Day7-11 [HG-U133B] | | |  |
| GSM565349 | -0.78 | -4.20(8) | Monocyte-Day1-6 [HG-U133B] | | |  |
| GSM565352 | -0.78 | -4.20(8) | Monocyte-Day1-9 [HG-U133B] | | |  |
| GSM565339 | -0.80 | -4.84(9) | Monocyte-Day0-8 [HG-U133B] | | |  |
| GSM565351 | -0.80 | -4.84(9) | Monocyte-Day7-2 [HG-U133B] | | |  |
| GSM565367 | -0.80 | -4.84(9) | DendriticCell-Control-6 [HG-U133B] | | | |
| GSM565334 | -0.83 | -6.19(11) | Monocyte-Day0-5 [HG-U133A] | | |  |
| GSM565328 | -1.00 | -3.54(5) | PlasmaCell-FromBoneMarrow-3 [HG-U133A] | | | |
| GSM565368 | -1.00 | -3.54(5) | DendriticCell-Control-1 [HG-U133B] | | | |
| GSM565311 | -1.00 | -3.54(5) | Bcell-naive-6 [HG-U133A] | | |  |
| GSM565369 | -1.00 | -4.27(6) | DendriticCell-Control-6 [HG-U133A] | | | |
| GSM565348 | -1.00 | -5.01(7) | Monocyte-Day1-7 [HG-U133B] | | |  |
